# *Lobelia siphilitica* Plants That Escape Herbivory in Time Also Have Reduced Latex Production

**DOI:** 10.1371/journal.pone.0037745

**Published:** 2012-05-25

**Authors:** Amy L. Parachnowitsch, Christina M. Caruso, Stuart A. Campbell, André Kessler

**Affiliations:** 1 Department of Ecology and Evolutionary Biology, Cornell University, Ithaca, New York, United States of America; 2 Department of Integrative Biology, University of Guelph, Guelph, Ontario, Canada; 3 Department of Entomology, Cornell University, Ithaca, New York, United States of America; Centro de Investigación y de Estudios Avanzados, Mexico

## Abstract

Flowering phenology is an important determinant of a plant’s reproductive success. Both assortative mating and niche construction can result in the evolution of correlations between phenology and other reproductive, functional, and life history traits. Correlations between phenology and herbivore defence traits are particularly likely because the timing of flowering can allow a plant to escape herbivory. To test whether herbivore escape and defence are correlated, we estimated phenotypic and genetic correlations between flowering phenology and latex production in greenhouse-grown *Lobelia siphilitica* L. (Lobeliaceae). *Lobelia siphilitica* plants that flower later escape herbivory by a specialist pre-dispersal seed predator, and thus should invest fewer resources in defence. Consistent with this prediction, we found that later flowering was phenotypically and genetically correlated with reduced latex production. To test whether herbivore escape and latex production were costly, we also measured four fitness correlates. Flowering phenology was negatively genetically correlated with three out of four fitness estimates, suggesting that herbivore escape can be costly. In contrast, we did not find evidence for costs of latex production. Generally, our results suggest that herbivore escape and defence traits will not evolve independently in *L. siphilitica*.

## Introduction

The timing of flowering has significant effects on plant fitness [1], [2]. For outcrossing plants, flowering time determines the availability of both mates and pollinators. Plants that flower when mates or pollinators are scarce will have reduced seed set [3], [4]. Furthermore, flowering time can affect the probability of damage by herbivores that consume reproductive tissue, such as florivores and seed predators. Plants that flower when these herbivores are abundant can have reduced seed set [5], [6], [7]. Flowering time also determines the abiotic environment in which plants reproduce. For example, plants that flower prior to the onset of drought can have higher seed set than those that delay flowering [8], [9]. Similarly, alpine plants that produce flowering buds later can have higher fitness because they avoid frost damage [10]. Because of these effects on interactions between plants and their environment, flowering time is frequently under natural selection in the wild [2], [11].

Flowering time is likely to be correlated with other reproductive, functional, and life history traits for two reasons. First, plants assortatively mate by flowering time [12]. Plants that flower early are more likely to mate with other early bloomers, while plants that flower late are more likely to mate with other late bloomers. This mating pattern will increase genetic correlations between flowering time and other functional traits [13]. Second, the timing of flowering determines the environment experienced by any traits expressed after flower initiation [14], and by extension the strength and direction of natural selection on those traits [15]. This niche construction can result in the evolution of genetic correlations between flowering time and subsequently expressed traits [15]. Such correlations can result in fitness trade-offs that may constrain the rate of adaptation [16], as well as contribute to the evolution of syndromes (suites of correlated traits that increase fitness [17], [18]).

Flowering time is likely to be correlated with herbivore defence traits, particularly those expressed in reproductive tissue. As a niche construction trait, flowering time determines whether a plant’s reproductive structures are exposed to herbivores [5], [19]. If a plant escapes herbivory, then there should be selection against costly defence traits [20]. Such niche construction is predicted to result in the evolution of genetic correlations between flowering time and defence traits [15]. Specifically, genotypes that escape herbivory because of their flowering phenology should have reduced investment in defence traits [21], [22], [23]. Genetic correlations between flowering time and defence traits could also be inflated by phenological assortative mating [13]. This is because mates will have both similar flowering times and a similar level of investment in herbivore defence. Despite increased interest in defence syndromes [18], few studies have estimated correlations between flowering time and other herbivore defences [24], [25], [26]. However, life history traits such as flowering time are often better predictors of herbivore damage than physical and chemical defence traits, perhaps because life history traits have larger effects on herbivore preference and performance [27].

**Figure 1:**
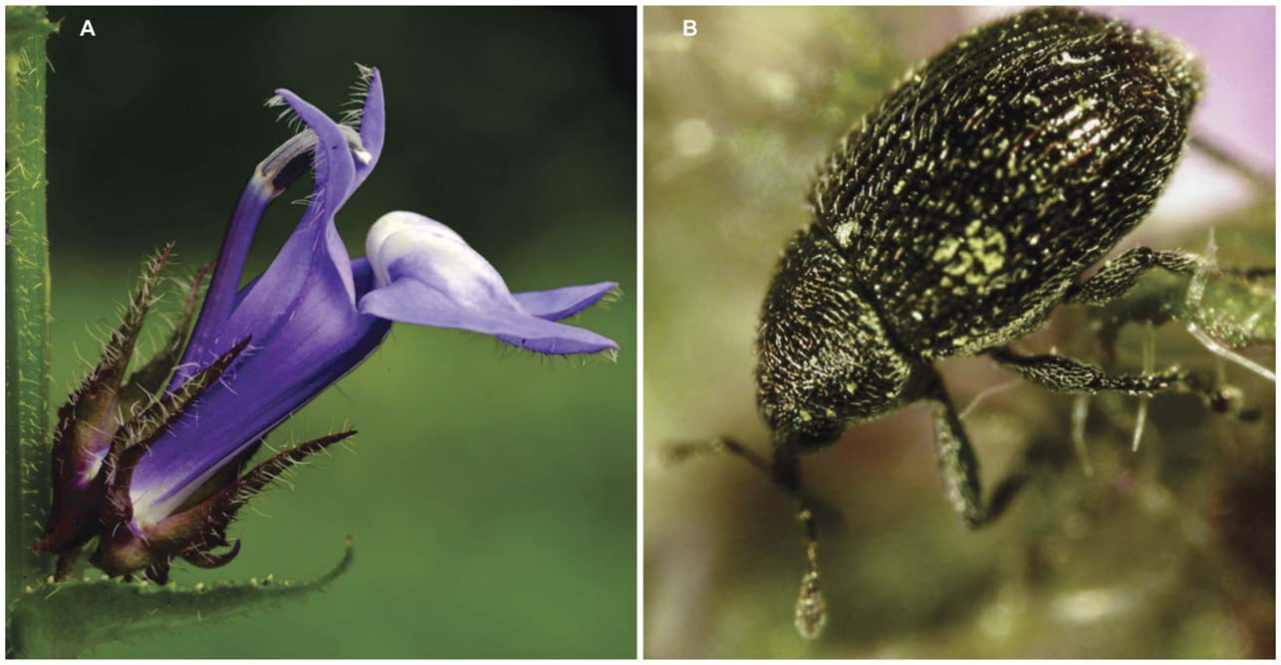
*Lobelia siphilitica* flower (A) and its specialist pre-dispersal seed herbivore, *Cleopmiarus hispidulus* (B). Photo credits: flower by Brian Husband, seed herbivore by Amy Parachnowitsch.

To test whether herbivore escape and defence are correlated, we estimated phenotypic and genetic correlations between flowering phenology and latex production in the wildflower *Lobelia siphilitica* L. (Lobeliaceae; Fig. 1A). This species is attacked by the specialist pre-dispersal seed herbivore *Cleopmiarus hispidulus* LeConte (Coleoptera: Curculionidae; Fig. 1B) [28]. In a previous study, Parachnowitsch and Caruso [5] found that late-flowering *L*. *siphilitica* escaped attack by *C*. *hispidulus*, resulting in direct natural selection for later flowering. However, *L*. *siphilitica* also produces an alkaloid-rich latex exudate [29], which likely acts as a defence against seed predation when exuded from the ovary walls [30]. Because late-flowering *L*. *siphilitica* escape attack by *C*. *hispidulus* there should be relaxed selection on defensive traits such as latex and/or alkaloid content in these plants. This niche construction, in combination with assortative mating, should result in the evolution of a negative genetic correlation between flowering time and latex production in *L*. *siphilitica*. Our prediction assumes that there are intrinsic fitness costs to delaying flowering and investing in latex production [31], [32] when herbivores are absent. However, costs of herbivore escape and defence are inconsistently observed [33].

We measured flowering phenology, latex production, and four fitness correlates (flower size, final plant height, rosette number and final biomass) of greenhouse-grown *L*. *siphilitica* in order to answer the following questions:

1. Are flowering time and latex production negatively phenotypically and genetically correlated in L. *siphilitica*, as expected if plants that escape herbivory have reduced investment in defence traits?
2. Are flowering time and latex production negatively correlated with flower size, final plant height, rosette number, and/or final biomass of L. *siphilitica*, as expected if herbivore escape and defence are costly?

## Results

There was significant phenotypic (Table 1) and genetic (Table 2) variation for flowering phenology, latex production, and fitness correlates of *L*. *siphilitica*. Day of first flower and latex production (wet and dry mass) varied significantly among maternal families. In addition, we detected effects of maternal family on all four fitness correlates (flower size, inflorescence height, rosette number and final biomass). The final position of the plant in the greenhouse (planting tray) also had a significant effect on flowering phenology, latex production (wet and dry mass), inflorescence height and final biomass, but not flower size or rosette number (Table 2).

**Table 1:** Summary statistics for the phenotypic measurements of greenhouse-grown *Lobelia siphilitica* plants.

Phenotypic traits	Mean	Range	*N*
Days to first flower	123	101–153	483
Wet latex mass (mg)	1.31	0–8.85	478
Dry latex mass (mg)	0.32	0–2.25	478
Flower size (mm)	12.00	9.89–14.91	483
Inflorescence height (cm)	61	28–106	477
Rosette number	10	0–25	477
Final biomass (g)	16.76	5.40–36.17	397

**Table 2:** Effects of maternal family and planting tray on variation in six phenotypic traits of greenhouse-grown *Lobelia siphilitica*.

Phenotypic traits	Maternal family	Planting tray
Days to first flower	*F*_23,454_ = 4.47****	*F*_93,454_ = 1.38*
Wet latex mass	*F*_23,451_ = 2.92****	*F*_93,451_ = 1.43*
Dry latex mass	*F*_23,451_ = 2.61****	*F*_93,451_ = 1.36*
Flower size	*F*_23,454_ = 2.00**	*F*_93,454_ = 1.13
Inflorescence height	*F*_23,450_ = 2.34***	*F*_93,450_ = 2.21****
Rosette number	*F*_23,450_ = 21.73*	*F*_93,450_ = 1.24
Final biomass	*F*_23,372_ = 3.45****	*F*_93,372_ = 1.63**

* *P*<0.05, ** *P*<0.01, *** *P*<0.001, **** *P*<0.0001.

**Figure 2:**
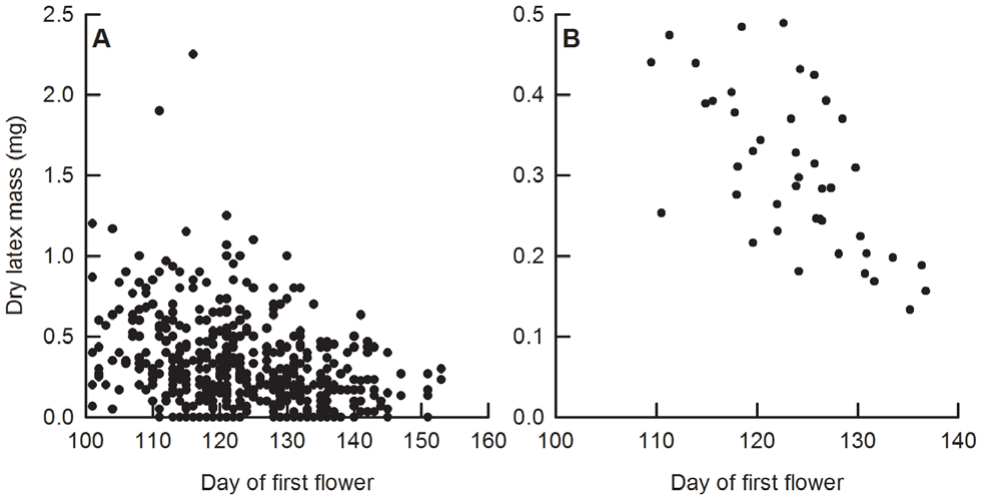
Relationship between flowering phenology and latex defence in greenhouse-grown *Lobelia siphilitica*. (**A**) Phenotypic correlation between days to first flower and dry latex mass (*N* = 478). (**B**) Maternal family mean correlation between days to first flower and dry latex mass (*N* = 46). Note the scales differ between panels to allow for better visualization.

Flowering phenology and latex production were both phenotypically and genetically negatively correlated (Table 3; Fig. 2). *Lobelia siphilitica* plants and families that flowered later produced significantly less latex, although the genetic correlation between wet latex mass and flowering phenology was not significant when corrected for multiple comparisons. Furthermore, the phenotypic correlation between phenology and latex remained significant after correcting for final biomass (wet latex mass: *r*_partial_ = −0.218, *P*<0.0001, *N* = 392; dry latex mass: *r*_partial_ = −0.257, *P*<0.0001, *N* = 392).

**Table 3:** Phenotypic and genetic correlations for six traits of greenhouse-grown *Lobelia siphilitica*.

Traits	Phenology	Wet latex	Dry latex	Flower	Height	Rosettes	Biomass
Phenology	**–**	−**0.30**	−**0.31**	−**0.17**	−**0.29**	−0.07	−**0.50**
Wet latex	−*0.34*	**–**	**0.74**	*0.13*	**0.14**	−0.01	**0.20**
Dry latex	−**0.56**	**0.89**	**–**	*0.11*	**0.20**	−0.03	**0.16**
Flower	−*0.31*	0.12	0.19	**–**	**0.17**	−0.04	0.10
Height	−*0.36*	0.14	0.22	*0.31*	**–**	−0.06	**0.35**
Rosettes	−0.09	0.06	0.01	0.15	−0.13	–	**0.26**
Biomass	−**0.54**	*0.30*	*0.40*	0.25	*0.41*	*0.31*	**–**

Phenology  =  days to first flower, Latex  =  wet or dry latex mass, Flower  =  flower size, Height  =  inflorescence height, Rosettes  =  rosette number, Biomass  =  final biomass. Phenotypic correlations are above and genetic correlations are below the diagonal. *N* = 392−483 for phenotypic correlations. *N* = 46 for genetic correlations. Correlations in **bold** are significantly (*P*<0.05) different from zero after Bonferroni correction by the Dunn-Šidák method. Correlations in *italics* were significant prior to but not after correction.

Correlations between flowering phenology and fitness estimates were generally negative in *L*. *siphilitica*. Early flowering plants produced larger flowers, taller inflorescences and accumulated a greater final biomass than late flowering *L*. *siphilitica* (Table 3). In contrast, day of first flower was not phenotypically correlated with rosette number. Of the three fitness estimates that were phenotypically correlated with day of first flower, only final biomass was significantly genetically correlated after Bonferroni correction.

In contrast to flowering phenology, latex mass (wet and dry) was not significantly negatively correlated with any of our four fitness estimates (Table 3). Instead, plants (but not families) that produced more latex had significantly taller inflorescences and greater final biomass. After correcting for day of first flower, the positive phenotypic correlation between latex mass and inflorescence length remained significant for dry (*r*_partial_ = 0.12, *P* = 0.009, *N* = 472) but not wet latex mass (*r*_partial_ = 0.06, *P* = 0.21, *N* = 472). However, the phenotypic correlation between latex mass and final biomass was not significant for either wet (*r*_partial_ = 0.07, *P* = 0.16, *N* = 392) or dry latex mass (*r*_partial_ = 0.01, *P* = 0.8, *N* = 392) after accounting for variation in phenology.

## Discussion

In our greenhouse population, *L. siphilitica* plants that flowered later and therefore would escape pre-dispersal seed herbivory in the field [5] also produced less latex (Fig. 2). This result is consistent with the prediction that niche construction [14] and assortative mating [12] will result in the evolution of correlations between flowering phenology and functional traits such as herbivore defence. The negative correlation between flowering phenology and latex production in *L*. *siphilitica* is also consistent with the prediction from optimal defence theory that plants which escape herbivory will invest less in herbivore defence [21], [22], [23]. More generally, our results suggest that herbivore escape and defensive traits do not evolve independently. Thus for plants where flowering time acts as an herbivore escape trait, phenology should be included in plant defence syndromes [27]. Likewise, the defensive function of flowering time may affect a plant’s reproductive strategy if herbivory is related to floral phenology.

We found a significant, genetically based negative correlation between temporal escape from herbivory and a putative herbivore defensive trait. Our results contrast with those of Juenger *et al.* [26] and Berenbaum, Zangerl & Nitao [24], who found that plants that escaped herbivory by flowering when herbivores were scarce produced more, rather than less, defensive chemistry against these herbivores. Genotype by environment interactions may explain the inconsistency of these three studies if correlations between herbivore escape and defence are seen in some environments but not others. However, flowering time may also be correlated with defence for reasons unrelated to their effects on herbivores suggesting that further examination of these traits in the field is necessary to determine their functional significance. For example, Johnson *et al.* [25] also detected a negative genetic correlation between flowering time and a defensive secondary compound in *Oenothera biennis*, but flowering time does not act as an herbivore escape trait for this species.

Escape in time has been documented in other species [34], [35], but this study demonstrates that such escape can be costly in the absence of herbivores. Moreover, our estimate of the cost of herbivore escape in time for *L*. *siphilitica* reflects only resource-based trade-offs (“direct costs” [32]). Costs can also arise from interactions with the biotic or abiotic environment (“ecological costs” [32]). Such ecological costs of delayed flowering are particularly likely in *L*. *siphilitica* because it flowers in the late summer and early fall. Consequently, plants that flower later may not have adequate time to mature seeds before the onset of winter. Although delayed flowering may also be costly because pollinators can be scarce later in the season [36], reproduction of late-flowering *L*. *siphilitica* was not pollen-limited in our source population [5] suggesting that pollination may not impose a significant ecological cost. More generally, our results suggest that in addition to the direct and ecological costs of chemical defence traits [37], traits that allow plants to escape herbivory in time may also carry costs.

In contrast to flowering phenology, we found no evidence that latex production was costly in *L*. *siphilitica*. Although defence theories generally assume costs to herbivore defence [38], ours is one of many studies that have not detected them [33]. There are three potential reasons why we did not detect costs in our greenhouse population. First, any negative correlations between latex production and our fitness estimates may have been masked by variation in resource acquisition [31]. This is because plants with high resource acquisition ability will have both high latex production and increased fitness. In particular, because the alkaloids in *Lobelia* spp. latex are rich in nitrogen, costs of latex production may only be detected in N-limited field soils. Second, given that *L*. *siphilitica* pollen is also nitrogen-rich, direct costs of latex production may be expressed through male fitness [32]. Although we did not estimate correlates of male fitness, we did find that female *L*. *siphilitica* plants, which by definition do not incur male fitness costs, had 64% higher dry latex production than hermaphrodites (unpaired *t*-test assuming unequal variances; *t* = 2.413, df = 15, *P* = 0.029). Finally, latex production may incur ecological rather than direct costs [32], which we could not measure in the greenhouse.

Latex has evolved multiple times in the angiosperms and is a key innovation in some clades, but little is known about its evolutionary ecology [30]. We found that there was significant genetic variation for latex production in *L*. *siphilitica* (Table 2). The only other study [39] that estimated quantitative genetic parameters for latex production also detected significant genetic variation for this trait, suggesting that it could evolve in response to selection by herbivores. However, Agrawal [39] found that herbivore-mediated selection for increased latex production was weak. Measuring selection on latex production in species such as *L*. *siphilitica* could indicate whether this weak relationship between latex and fitness is common.

Our studies on *L. siphilitica* suggest that changes in flowering phenology can not only affect interactions with herbivores, but may also alter natural selection on defences. Specifically, any herbivore-mediated selection for later flowering in *L*. *siphilitica* should result in indirect selection for reduced latex production. Although flowering phenology has often been considered only in terms of its ecological and evolutionary effects on interactions for pollinators [1], our study adds to the growing interest in the relationship between flowering time and herbivory [27], [40], [41]. Understanding which traits are likely to co-evolve with phenology is particularly important in the face of shifts in flowering phenology under a changing climate [42]. Natural selection imposed by changes in climate can result in the rapid evolution of flowering time [43], as well as traits that are genetically correlated with flowering time [44]. Thus, for species where flowering phenology is related to herbivore attack, shifts in flowering time due to climatic change could also indirectly shape the evolution of herbivore defence.

## Materials and Methods

### Study System

*Lobelia siphilitica* is a short-lived, herbaceous perennial native to eastern North America. It reproduces sexually by a single racemose inflorescence, although some individuals produce additional lateral inflorescences. It also reproduces asexually by producing secondary rosettes. *Lobelia siphilitica* is self-compatible but cannot autonomously self-fertilize, making pollinators essential for seed set [45]. In Ontario, Canada, plants flower from late July into September and fruits ripen from September to early October [5]. Although *L. siphilitica* is gynodioecious [46], female plants are rare in the northern part of its range [47].

*Lobelia siphilitica* produces an alkaloid-rich latex exudate from the leaves, stems and ovary walls [29], which likely functions as a plant defence [30]. Latex is considered one of the most efficient defences against herbivores because it both physically gums up insect mouth parts and contains defensive chemical compounds [30]. Female *C. hispidulus* are exposed to *L*. *siphilitica* latex when they bore a hole through the wall of the ovary with their mandibles before laying an egg inside the ovary (A. L. Parachnowitsch, personal observation). We have observed little foliar herbivory (A. L. Parachnowitsch & C. M. Caruso, personal observation) but high rates of pre-dispersal seed herbivory in the source population used for this study (89–92.5% in 2003–4) [5], [48], suggesting that *C*. *hispidulus* may be an important agent of selection on latex production.

Two lines of evidence suggest that latex is an effective defence in *L*. *siphilitica*. First, lobeline, a chemical compound found in *Lobelia* spp. latex, elicits trenching in Noctuid caterpillars [49]. Trenching is a typical latex avoidance behaviour [30] that significantly reduces leaf alkaloids in *Lobelia cardinalis* [50], as expected if latex functions as a defence against insect herbivores. Second, when *C*. *hispidulus* oviposits on *L. siphilitica* flowers, latex exudes out of the wound on the ovary wall. However, many of these wounds do not have an egg (A. L. Parachnowitsch, personal observation), suggesting that latex can prevent *C*. *hispidulus* from successfully attacking *L*. *siphilitica* fruits.

### Study Design

The seeds used for our study were offspring of *L*. *siphilitica* included in a field experiment designed to estimate the strength of phenotypic selection on flowering phenology and identify the agents of this selection [5]. To generate plants for this field experiment, we collected open-pollinated seeds (hereafter grand-maternal families) from a *L*. *siphilitica* population near Guelph, Ontario, Canada. No specific permits were required for their collection. The seeds were grown to flowering in the greenhouse upon which plants were returned to their source population, where they were also open-pollinated. We selected 46 of these open-pollinated maternal families, two from each of 23 grand-maternal families. Given this design, offspring of each maternal family were half- or full-siblings and the offspring within each grand-maternal family were cousins.

We rinsed seeds in a distilled water, bleach and ethanol solution (16∶1:1) to break dormancy [46]. All seeds from each fruit were germinated on wet filter paper in Petri dishes for two weeks and the day of first germination for each dish was recorded. To ensure that we had enough seedlings, we germinated seeds from two fruits per maternal family. If both dishes had successful germination, we chose the fruit that began germinating earlier. However, both fruits were used from two of the maternal families to ensure that we had enough seedlings. We transplanted 24 seedlings per maternal family to 72-well plug trays on March 10, 2006 and replaced any dead seedlings after four days. Families and seedlings within families were randomly assigned to a position within trays and trays were rotated within the greenhouse weekly. Seedlings were grown for six weeks before transplanting 12 randomly chosen plants per maternal family to 10 cm diameter pots filled with greenhouse potting soil (Promix®). We randomly assigned plants to trays that were stationary for the remainder of the study and bottom-watered to maintain flooded soil conditions. Plants were treated for common greenhouse pests (thrips, whiteflies and fungi) approximately once per month and fertilised weekly until the majority of rosettes had begun bolting.

### Phenotypic Measurements

To determine if herbivore escape and defence were correlated in *L*. *siphilitica*, we measured flowering phenology and latex production. We censused plants daily to determine the day of first flower (hereafter flowering phenology). As each plant flowered, we collected latex from two flowers per plant to estimate latex production. When possible we sampled early-produced flowers because they are more likely to be attacked by *C*. *hispidulus* in the field [48]. Flowers were clipped at the base of the ovary with scissors and the latex exudate from the wound on the pedicel was collected on pre-dried and pre-weighed filter paper (Whatman’s No. 1) until the flow stopped [51]. Thus latex mass reflects latex flow to the entire flower, rather than the flow to a single weevil wound. However, the quantities to a single wound would be too small to detect with our methods. We weighed the latex-soaked filter paper both prior to and after drying at 60°C for at least 24 h to estimate wet and dry latex mass, respectively. Wet mass measures the overall amount of latex flow to a wound (physical defence) while dry mass estimates the chemical constituents of the latex. Latex effectiveness, either in toxicity or physical properties, was not estimated for these plants. Because flowers within a plant are not independent of each other, we used the mean latex exuded by the two flowers collected from each plant for all of our analyses.

To test whether herbivore escape and defence are costly, we non-destructively estimated three fitness correlates: flower size, inflorescence height, and rosette number [5], [52]. We measured petal width, petal length and corolla tube width [5] for at least five flowers per plant sampled from along the raceme. We then took the geometric mean of these three measurements [5], [53], [54] as an overall size estimate for each flower. We use the geometric mean rather than the first principle component (PC) of a PC analysis because the mean directly relates to size whereas the PC can introduce errors in interpretation [55]. Because flowers within a plant are not independent of each other, we used the mean flower size per plant for all of our analyses. When all plants had finished flowering, we measured inflorescence height and the number of rosettes produced. Inflorescence height is positively correlated with flower number [5] and previous studies indicate that *L. siphilitica* with larger flowers and taller inflorescences produce more seeds [5], [52]. However, the relationship between these traits and male fitness is unknown. Because *L*. *siphilitica* rosettes can overwinter and produce a flowering stalk in the following year [56], we interpreted rosette number to be a correlate of fitness via asexual reproduction.

When all plants had finished flowering (approximately six months from the start of germination), we destructively estimated final biomass as an additional fitness correlate. We clipped the inflorescence and any rosettes and dried them at 45°C for 24 h to measure aboveground biomass. To estimate belowground biomass, we washed, dried, and weighed the roots of a subset of the plants (*N* = 90) in the study. Initially, mass was estimated separately for the roots that were contained in the pot and those that emerged out of the pot into the water-filled tray. Because the mass of contained and emerged roots was positively correlated (*r* = 0.411, df = 89, *P*<0.0001), we estimated belowground biomass for the remaining plants based on the mass of their emerged roots (belowground root biomass  = 2.79+1.88× emerged root mass + emerged root mass). We estimated final biomass as the sum of the aboveground and estimated belowground biomass for each plant. This estimate of final biomass was strongly positively correlated with the sum of aboveground biomass and emerged root biomass (*r* = 0.976, df = 89, *P*<0.0001). We estimated final biomass, rather than biomass at the initiation of flowering, because we were interested in the consequences of delayed flowering in *L*. *siphilitica*, rather than the causes of delayed flowering (for example, small vegetative size [57]). Although the overall length of growing time was similar between the greenhouse and field conditions, greenhouse plants were not pollinated and were protected from end of growing season frosts. Consequently, we may underestimate fitness costs of delayed flowering.

Data deposited in the Dryad Repository: http://dx.doi.org/10.5061/dryad.n3f4g.

### Statistical Analysis

Our final data set (*N* = 483 plants) was unbalanced, with 7–12 offspring per maternal family. We eliminated 16 female plants plus one unsexed plant from our data set because female *L. siphilitica* can differ phenotypically from hermaphrodites [52], [58]. In addition, 48 plants died prior to the end of the study and four plants were excluded because the day of first flower was not recorded. Finally, some traits were not measured on all plants and thus had an *N* <483.

Prior to analyzing our data, we used ANOVA to test whether germination time differed between families. Germination time did not differ significantly among grand-maternal families (*F*_22,86_ = 2.10, *P* = 0.09) or nested maternal families (*F*_21,86_ = 1.94, *P* = 0.11). This suggests that, consistent with studies in other species [59], variation in date of first flower is independent of variation in germination time in *L*. *siphilitica*. Consequently, we did not include germination time as a covariate in our analyses.

We also used ANOVA to test whether there was a genetic basis to variation in flowering phenology, latex production, and our fitness correlates (flower size, inflorescence height, rosette number, and final biomass). Our model included terms for grand-maternal family and maternal family nested within grand-maternal family. In addition, we included a term for greenhouse position (the final randomly assigned planting tray) to control for any effect of location in the greenhouse on phenotype. Because the maternal families were also greenhouse reared, they are likely less biased by environmental maternal effects than the grand-maternal families. Therefore, if the term for maternal family was significant, we concluded that there was a genetic basis to variation in that trait.

We estimated phenotypic correlations among flowering phenology, latex production and fitness estimates (flower size, inflorescence height, rosette number, and final biomass) as the Pearson correlation coefficient. To test whether there was a genetic basis to these phenotypic correlations, we estimated genetic correlations as the Pearson correlation coefficient among maternal family means. Family mean correlations can be biased estimates of the true genetic correlation [60]. However, for our data set family mean correlations were similar in magnitude and significance to genetic correlations estimated using restricted maximum likelihood approaches (data not shown), suggesting that our conclusions are robust to the estimation technique used. We maintained an experiment-wide error rate of α = 0.05 for each matrix of correlations using the sequential Bonferroni correction by the Dunn-Šidák method [61].

Correlations between herbivore defence or escape traits and general vigour may obscure the relationship between these traits [31]. Therefore, we used partial Pearson correlations controlling for final biomass to assess whether the relationship between flowering phenology and latex production was independent of overall size. If variation in general vigour was driving the relationship between escape and defence in *L*. *siphilitica*, then these partial phenotypic correlations would be non-significant.

Furthermore, the cost of latex production may have been masked by correlations with flowering phenology; larger plants had more latex and flowered earlier (Table 3). Thus, to explore this possibility, we controlled for variation in flowering phenology by estimating the partial Pearson correlation between latex production and the fitness estimates. If variation in phenology was masking costs of latex production, then these partial phenotypic correlations would be negative rather than positive.

Four features of our design could have inflated our estimates of genetic variation and genetic correlations. First, because we have maternal rather than paternal families, our estimates of these genetic parameters include not only additive genetic variance, but also common maternal effects. If common maternal effects are substantial, then our estimates will be inflated relative to estimates of genetic parameters calculated from paternal family designs [60]. Second, because we germinated seeds from open-pollinated plants, our families consist of an unknown mixture of full- and half-siblings. Consequently, our estimates of genetic variation and genetic correlations may include dominance genetic variance in addition to additive genetic variance [60]. Third, our open-pollinated families could have included offspring produced through geitonogamous self-pollination. Such inbreeding is expected to decrease the standing genetic variation within populations [62]. Fourth, we measured genetic variation and genetic correlations for plants grown in a greenhouse environment. Greenhouse estimates of genetic variation for plant functional [63] and floral [64] traits are generally higher than field estimates and genetic correlations can be environmentally dependent [65]. However, the one study to compare greenhouse and field estimates of genetic correlations found that they can be quite concordant, at least relative to estimates of genetic variation [64]. In addition, because costs can only be estimated in the absence of herbivory, they are often measured in greenhouse conditions [66], making our estimates of the costs of escape and a putative defensive trait comparable to many other studies.
